# Vegetation Composition of the Halophytic Grass *Aeluropus lagopoides* Communities within Coastal and Inland Sabkhas of Saudi Arabia

**DOI:** 10.3390/plants11050666

**Published:** 2022-02-28

**Authors:** Basharat A. Dar, Abdulaziz M. Assaeed, Saud L. Al-Rowaily, Abdullah A. Al-Doss, Ahmed M. Abd-ElGawad

**Affiliations:** 1 Plant Production Department, College of Food & Agriculture Sciences, King Saud University, P.O. Box 2460, Riyadh 11451, Saudi Arabia; bdar@ksu.edu.sa (B.A.D.); assaeed@ksu.edu.sa (A.M.A.); srowaily@ksu.edu.sa (S.L.A.-R.); aaldoss@ksu.edu.sa (A.A.A.-D.); 2 Department of Botany, Faculty of Science, Mansoura University, Mansoura 35516, Egypt

**Keywords:** phenotypic plasticity, mangrove grass, salt marshes, coastal habitat, biodiversity

## Abstract

Sabkhas are unique, highly saline ecosystems, where specially adapted plants can grow. *Aeluropus lagopoides* (L.) Thwaites is a halophytic forage plant growing in salt marsh habitats of inland and coastal sabkhas of Saudi Arabia. The present study provides an analysis of vegetation composition and distribution of the *A. lagopoides* community in five different regions within Saudi Arabia, emphasizing the environmental factors that affect species distribution. The floristic survey revealed the presence of 48 species, belonging to 26 families. Poaceae, Chenopodiaceae, Mimosaceae, Zygophyllaceae, and Asteraceae are the largest families (50% of total species). Phanerophyte, followed by chamaephytes, are the most frequent forms, indicating a typical saline desert life-form spectrum. The vegetation analysis revealed the dominance of *A. lagopoides* in all locations, where it was the most dominant species in Qareenah, Qaseem, and Salwa locations, and the second most dominant species in Jouf and Jizan locations. The flourishment of this halophytic grass within a wide soil range in sabkhas revealed its adaptability to the harsh environment, which could be ascribed to its structural adaptations and modifications, as well as the phenotypic plasticity. The Qareenah and Qaseem locations attained the highest species richness and evenness, while the Jizan location was the least diverse. Within the studied locations, other highly salt-tolerant species were determined with high abundances, such as *Suaeda aegyptiaca* (Hasselq.) Zohary, *Zygophyllum album* L.f., *Tamarix nilotica* (Ehrenb.) Bunge, *Cressa cretica* L., and *Salicornia europaea* L. The soil analysis showed a significant variation for all parameters among the studied locations, except for pH, chloride, and clay content. The Qaseem location revealed the highest values of most soil parameters, while the Jizan location showed the lowest. The canonical correspondence analysis (CCA) showed that the community structure and diversity are mainly affected by the soil salinity and moisture. Due to the economic potentialities of *A. lagopoides* as a forage plant and sand stabilizer, the conservation of its habitats is of vital importance. In addition, this grass could be integrated as a promising forage candidate that can be planted in saline-affected areas, even in the summer dry season.

## 1. Introduction

Sabkhas are geological phenomena formed in an arid or semi-arid climate, as broad plains or salt flats, containing evaporates dictated by the local water table [1]. Geographically, it has a large habitat range, distributed worldwide through Southeast Europe, the siliciclastic coast of California, Mexico, North Africa from Morocco to Somalia, the Middle East and the Arabian Peninsula, Australia, and Asia [2,3,4]. Sabkhas are grouped into two major landform types [5], low-lying coastal salt marshes [6,7] or inland interdune areas as salt-crusted depressions [8]. Nearly all key species in these saline habitats are perennial halophytes forming different plant communities [4,9].

The Arabian Peninsula is also characterized by high salinity, high annual temperature variations, and shallow waters. Saudi Arabia possesses harsh natural desert environments without rivers or lakes. It is distinguished by various ecosystems, including mountains, wadis, meadows, rocky mountains, sandy deserts, and saltpans with distinct plant communities [10,11]. The salt-affected areas of Saudi Arabia are classified into the coastal plain, inland zone, and littoral salt marshes [12]. Coastal and inland saline habitats, called sabkhas, are highly stressful environments, as they are highly saline and wet unique ecosystems, where specially adapted halophytic plants can grow [13]. These sabkhas are mostly saturated with brine, and the soil surface is often encrusted with thick salt crust [14]. Natural saline habitats vary in salinity levels due to differences in topography, soil properties, and micro-climate, both spatially and temporally [15]. Vegetation composition in these ecosystems is influenced by complex heterogonous environmental factors, including duration and degree of inundation by seawater and both overground and underground freshwater input [16], coastal and inland geomorphology, microtopography, soil moisture content, and soil type [17].

Specific plant species can dominate sabkha habitats, forming monospecific stands. The vegetation of these monospecific stands forms zones with distinct plant communities [18], forming a variety of specialized habitats of distinct vegetation mosaics [19]. Most of the key species in the saline habitats are perennial halophytes, which constitute about 2% of the world’s flora [18,20], predominantly belonging to the families Chenopodiaceae, Zygophyllaceae, Plumbaginaceae, Poaceae, and Juncaceae [21]. The distribution of some halophytic species is best correlated along a gradient of soil variables, such as salinity, moisture content, soil texture, organic matter, and calcium carbonate [22].

Among the Poaceae family, *Aeluropus lagopoides* (L.) Thwaites is one important key species of saline habitats of Saudi Arabia [23]. Geographically, it has a wide habitat range, distributed through Southeast Europe, North Africa, the Middle East, Arabian Peninsula, and Central Asia [3]. In Saudi Arabia, *A. lagopoides* grows in various coastal and inland sabkhas [23,24]. It is restricted in the form of specialized vegetation patches in the Wadi Hargan, Riyadh, salt marsh sabkha of the Qaseem and Jouf, and coastal zones of the Salwa and Jizan regions [25,26]. It is of economic importance, where it is utilized as fodder in arid areas, used to stabilize sand dunes [27], and can be used for landscaping of the urban areas [28].

Knowledge of the ecological distribution of key species is the primary characteristic of its conservation strategy in its ecosystem [29]. Apart from the establishment and maintenance of the protected area, baseline information about the key species, vis a vis the associated species, edaphic factors of the habitats, environmental variation, anthropogenic activities of the regions, are crucial [25,30]. This ecogeographical survey is considered central to all the conservation issues and a key requisite in the development of the conservation strategy [31,32]. According to our field observations, *A. lagopoides* has various phenotypic characteristics and forms distinct vegetative patches within various regions of Saudi Arabia. To the best of our knowledge, no study has dealt with the vegetation composition of the key forage halophyte *A. lagopoides* in Saudi Arabia. Therefore, the present study aims to assess the vegetation structure of *A. lagopoides* communities in the various regions/habitats around Saudi Arabia, considering regional heterogeneity, edaphic factors, and variation of climatic gradients. This study will help in understanding the distribution of this important forage plant that flourished in one of Earth’s harsh environments and, in consequence, shows the potential of conserving this plant, as well as integrating it in a foraging system.

## 2. Results

### 2.1. Floristic Composition of the Studied Regions

As expected, the plant diversity of the studied sabkha regions is low, in which the species had to withstand harsh environmental conditions, i.e., the high salinity content (Table S1). The floristic analysis revealed the presence of 48 species of vascular plants, which are mainly perennials (75%). The highest number of species (24 species: 22 perennials and 2 annual) were recorded in the inland Sabkha of Qareenah, Riyadh region, which is represented by about 34% of the total recorded species, while the sabkha of the northern Al-Jouf region recorded the lowest number of species (all eight perennial species) which is about 10% of the total recorded species (Figure S1). However, the species’ evenness was highest in the inland sabkhas of the Qaseem region, representing about 42% of the recorded species, and the lowest was in the coastal sabkha of Jizan, representing about 9% (Figure S1). Summing up, the coastal sabkhas (Salwa and Jizan) recorded the highest number of species (43 species: 36 perennials and 6 annuals), which is represented by 60% of total recorded species, compared to the inland sabkhas (Qareenah, Qassem, and Jouf) that represented 40% of the total recorded species (29 species: 21 perennials and 8 annuals).

**Figure 1:**
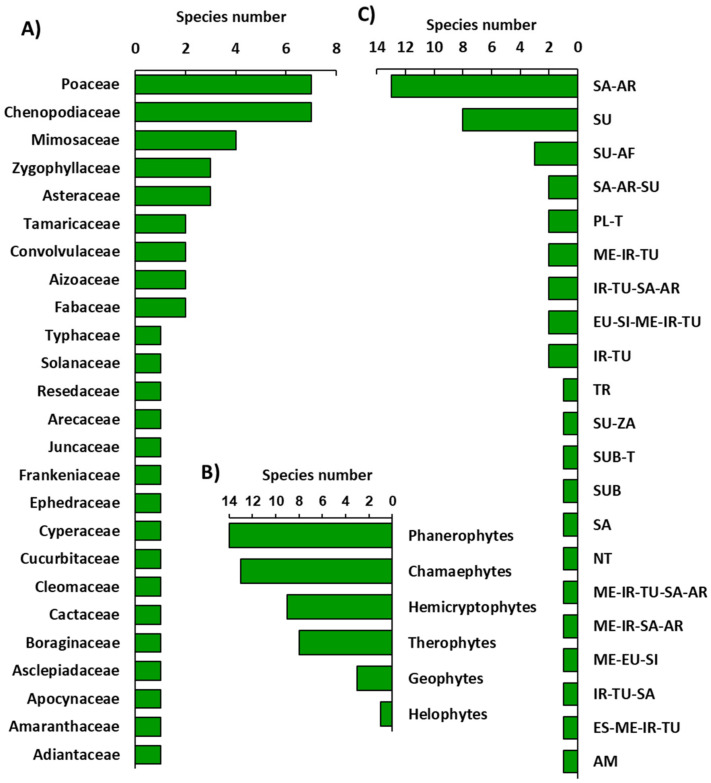
Floristic composition of the surveyed region. (**A**) Represented families, (**B**) life forms, and (**C**) chorotype spectra. SA-AR: Saharo-Arabian, SU: Sudian, AF: African, ME: Mediterranean, IR-TU: Irano-Turanian, EU-SI: Euro-Siberian, PL-T: Plurireginalbor-trop, SU-ZA: Sudano-Zambezian, SUB-T: Subtropical-Tropical, SA-South-American, NT-Neotropical, AM-American.

The identified plant species belonged to 26 families, where Poaceae, Chenopodiaceae, Mimosaceae, Zygophyllaceae, and Asteraceae were the major families that represented 50% of the total species (Figure 1A and Table S1).

On the one hand, The recorded plant species were classified into six life forms, according to Raunkiaer’s system, of which 29% were phanerophytes and 27% chamaephytes, while hemicryptophytes, geophytes, and helophytes were represented by 19%, 17%, and 2%, respectively (Figure 1B). On the other hand, the chorological analysis of the identified species revealed that 62.50% of those species were monoregional, and the Saharo-Arabian element was the most present chorotype (27.08%) (Figure 1C). However, 22.92% of the identified species were classified as biregional plants, whereas Sudanian-African was the most represented element (6.25%). The pluriregional chorotype was also represented, with 14.58% of the total recorded species, and the most represented ones were the Euro-Siberian-Mediterranean-Irano-Turanian and Saharo-Arabian-Mediterranean-Irano-Turanian, which represented 4.17% each.

### 2.2. Vegetation Analysis of the Studied Regions

The analysis of importance values of each species, based on the relative cover and density, led to the recognition of the dominant and important species within each location (Table 1). The details of all species are presented in Table S2. The Qareenah region was the most diversified, with 24 species. This group attained the highest richness (Simpson diversity index = 0.95). In this location, *A. lagopoides* was the first dominant species (importance value = 44.41), while *Zygophyllum coccineum* L. was the second most dominant (importance value = 25.64). The other important species recorded were *Juncus rigidus* Desf., *Tamarix nilotica* (Ehrenb.) Bunge, *Rhazya stricta* Decne., *Acacia gerrardii* Benth., and *Phragmites australis* (Cav.) Trin. ex Steud. (Table 1).

The location of Qaseem is dominated by *A. lagopoides* (importance value = 94.20). In this location, *Suaeda aegyptiaca* was determined as the second most dominant species (importance value = 28.61). The other important species that attained high importance values were *Cressa cretica* L., *J. rigidus*, *Lycium shawii* Roem. and Schult., and *Salicornia europaea* L. This location attained a Simpson diversity index of 1.6 and Shannon evenness of 0.74. On the other hand, the Salwa location showed the lowest Simpson diversity index (1.04), and it was dominated by *A. lagopoides* (importance value = 66.62), followed by *Zygophyllum album* L.f. (importance value = 41.05). The other important species within this location were *P. australis*, *J. rigidus*, *S. aegyptiaca* (Hasselq.) Zohary, and *Phoenix dactylifera* L. (Table 1).

The Jouf location was the least diversified (eight species) among the recognized groups, and it is dominated by *T. nilotica* as the most dominant and *A. lagopoides* as second most dominant species. This group attained a Simpson diversity index of 1.53 and Shannon evenness of 0.75. The other important species recorded in this group were *Z. album*, *C. cretica*, and *S. aegyptiaca* (Table 1). Lastly, the Jizan location comprised 15 recorded species. This community attained a Simpson diversity index of 1.81 and Shannon evenness of 0.75. The most dominant in this location was *S. aegyptiaca* (importance value = 80.76), while *A. lagopoides* was the second most dominant species. The other important species of this group were *Panicum repens*, *Cyperus conglomeratus* Rottb., *Aerva javanica* (Burm.f.) Juss. ex Schult., and *Zygophyllum simplex* L. (Table 1). *A. lagopoides* dominated in all studied locations, either as most dominant, as in the inland sabkhas of Qareenah, Qaseem regions, and Coastal sabkhas of Salwa, or second most dominant species, in the inland sabkhas of Jouf and coastal sabkha of the Jizan region (Table 1).

**Figure 2:**
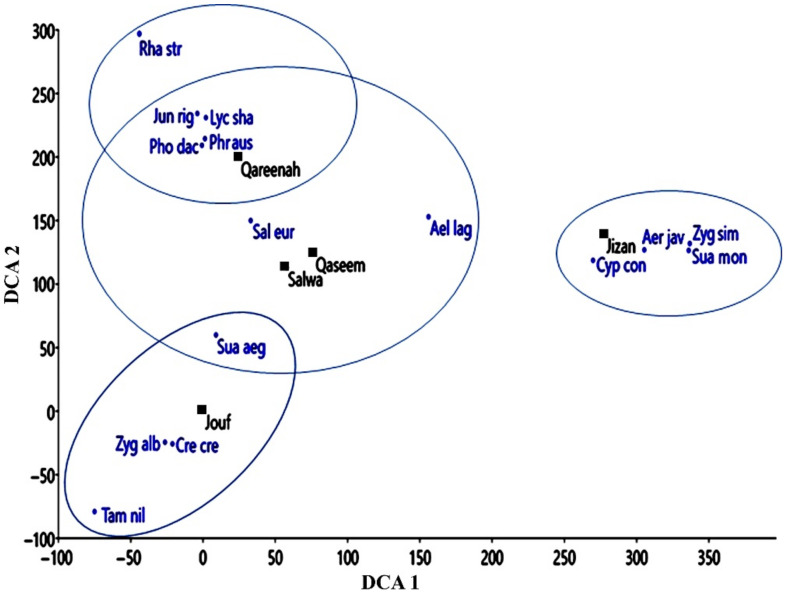
Detrended correspondence analysis (DCA) ordination of the studied locations (■) based on the importance value of dominant, co-dominant, and important species (●) recorded from each location. Ael lag: *Aeluropus lagopoides,* Tam nil: *Tamarix nilotica*, Jun rig: *Juncus rigidus*, Lyc sha: *Lycium shawii*, Pho dac: *Phoenix dactlifera*, Phr aus: *Phragmites australis*, Sal eur: *Salicornia europaea*, Sua aeg: *Suaeda aegyptiaca*, Zyg alb: *Zygophyllum album*, Cre cre: *Cressa cretica*, Cyp con: *Cyperus conglomeratus*, Zyg sim: *Zygophyllum simplex*, Aer jav: *Aerva javanica*, Pan rep: *Panicum repens*, Sau mon: *Suaeda monoica,* Rha str: *Rhazya stricta*.

The application of detrended correspondence analysis (DCA) on the vegetation data showed the separation of the Jizan location, on the right side of the DCA diagram (Figure 2). However, the other location showed quite significant overlapping, with a close correlation between the Qassem and Salwa locations.

**Figure 3:**
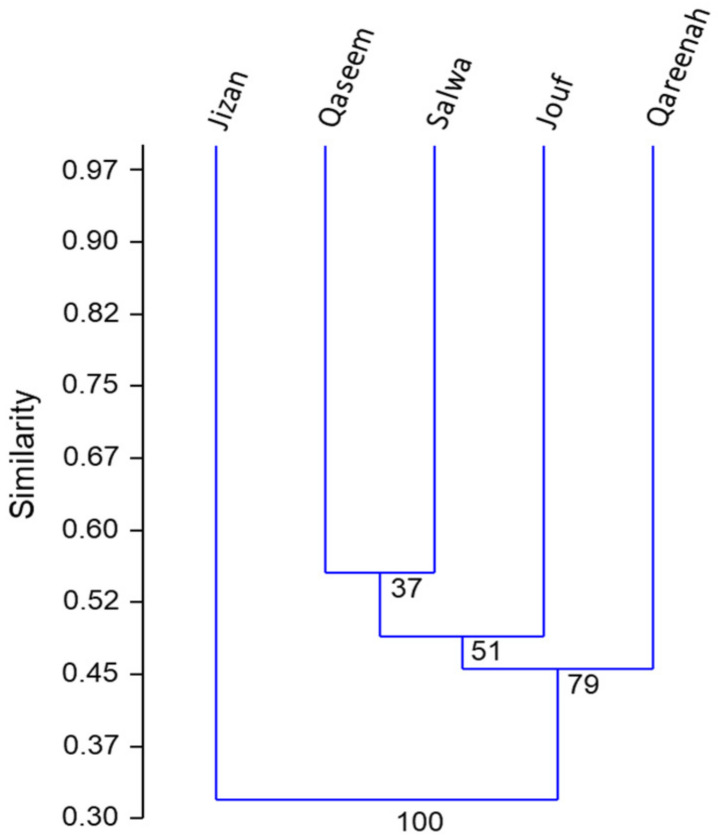
Hierarchical clustering of different studied locations based on the importance values of the recorded plant species (n = 48).

Moreover, the cluster analysis of the vegetation data of all recorded species confirmed the data of DCA, where it revealed that the Jizan location is different than other locations (Figure 3). Salwa and Qaseem locations showed similar vegetation composition, while Jouf and Qareenah were different in the vegetation structure.

### 2.3. Vegetation-Soil Relationship

The soil analysis of the five studied locations showed significant variations regarding all measured parameters, except for pH, Cl^−^, and clay (Table 2). The Qaseem location attained the highest moisture, clay, silt, salinity, Ca, Mg, Na, K, Cl, SO_4_, HCO_3_, and organic matter (Table 2). The Salwa location is characterized by high sand content, while the soil of the Qareenah location revealed the highest content of calcium carbonate. The Jizan location had the lowest moisture content, pH, organic matter, K, sulphate, bicarbonate, and calcium carbonate, while the Jouf location attained the lowest salinity content.

**Figure 4:**
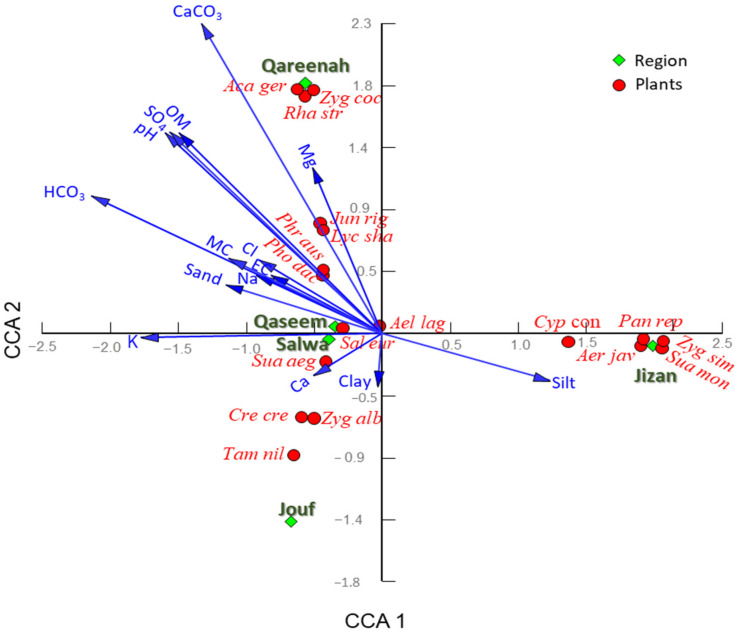
Canonical correspondence analysis (CCA) showing the correlation between the soil variables and dominant and important species representing the studied locations. Aca ger: *Acacia gerrardii*, Ael lag: *Aeluropus lagopoides*, Zyg coc: *Zygophyllum coccineum*, Sua aeg: *Suaeda aegyptiaca*, Zyg alb: *Zygophyllum album*, Tam nil: *T. nilotica*, Jun rig: *Juncus rigidus*, Rha str: *Rhazya stricta*, Phr aus: *Phragmites australis*, Cre cre: *Cressa cretica*, Lyc sha: *Lycium shawii*, Sal eur: *Salicornia europaea*, Pho dac: *Phoenix dactlifera*, Pan rep: *Panicum repens*, Cyp con: *Cyperus conglomeratus*, Aer jav: *Aerva javanica*, Zyg sim: *Zygophyllum simplex*. OM: organic matter, EC: electrical conductivity.

The correlation between the vegetation composition and soil properties was assessed by canonical correspondence analysis (CCA). The CCA showed that inland sabkhas of the Qareenah region were separated on the upper left side of the CCA biplot and showed a close correlation to CaCO_3_, Mg, organic matter content, pH, and sulphate (Figure 4). In contrast, the inland sabkha of the Jouf region is segregated in the CCA biplot’s lower left side, where they are affected by Ca and Clay. On the other hand, the coastal sabkha of the Jizan region was segregated on the lower right side of the CCA biplot, where it showed a correlation to silt contents. Finally, the inland sabkha of Qaseem and coastal sabkha of Salwa were separated on the central part of the CCA biplot, where they showed a positive correlation with salinity, moisture, K, bicarbonates, Na, and sand (Figure 4).

**Figure 5:**
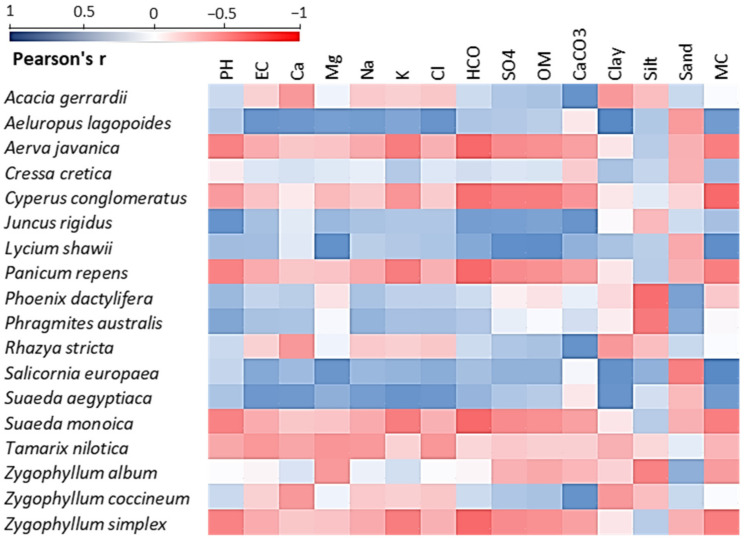
Pearson’s correlation heatmap between the soil variables and the dominant, co-dominant, and important associated plant species within the studied locations. EC: electrical conductivity, OM: organic matter, and MC: moisture content.

The Pearson’s correlation analysis between soil variables and dominant, co-dominant and important species is shown in Figure 5. *A. lagopoides*, the most dominant species of the Qareenah, Qaseem, and Salwa regions, and second most dominant species of the Jouf and Jizan regions, showed a strong positive correlation to all tested soil parameters, except for CaCO_3_ (r = −0.07) and sand (r = −0.54). Similarly, *S. aegyptiaca* (the most dominant species of the Jizan region and the second most dominant species of the Qaseem region), *J. rigidus*, *L. shawii*, *P. dactylifera*, *P. australis,* and *S. europaea*, revealed a positive correlation for all tested characteristics, except for CaCO_3_ and sand contents.

However, *T. nilotica*, the most dominant species of the Jouf Region, was negatively correlated with all the soil parameters, except for sand (r = 0.20). *Z. coccineum*, the second most dominant species of the Qareenah region, showed a negative correlation with most of the soil parameters, except for pH (r = 0.35), anions, and sand (r = 0.36). Among the important associated species, *J. rigidus*, *L. shawii*, *P. dactylifera*, *P. australis,* and *S. europaea* showed a strong positive correlation with almost all the soil parameters (Figure 5).

## 3. Discussion

Saudi Arabia is located within an arid and semi-arid zone. It is distinguished by its different ecosystems, including mountains, meadows, valleys, rocky and sandy deserts, and salt marshes [11,25]. Among salt marsh ecosystems, there are inland salt marshes and saline coastal habitats, called sabkhas. These sabkhas have scarce vegetation due to severe environmental conditions (wind exposure, high temperature, and high salinity). However, sabkha edges (transition towards sand) are characterized by well-defined zones, and each zone occupies a particular plant community. The salt-tolerant or halophytic plants, which constitute about 2% of the world’s flora [20], grow in these habitats. The spatial distribution of plants in these vegetation zones is affected by soil salinity and soil composition. This explained the low plant diversity in the studied sabkha locations.

The floristic analysis of this study revealed the predominance of phanerophytes and chamaephytes, reflecting the domination of perennial halophytes over annuals and ephemerals in saline conditions. The perennial halophytes can tolerate high salt content in soil [33]. The predominance of Poaceae, Chenopodiaceae, and Mimosaceae in the studied habitats was in harmony with other previous studies in different saline habitats [25,34]. On the other hand, most of the recorded species belong to the Saharo-Arabian element. This finding is in harmony with other studies on salt marsh habitats [10].

Based on the data of diversity indexes, the Qareenah location showed the highest richness. This could be ascribed to the water factor, as this location is a wadi system and has a relative amount of water. In our previous work, a total of 111 plant species were recorded within this wadi, even some hydrophytes and ferns are flourished [25]. The diversification of the Qareenah location could be ascribed to the fact that it forms a woodland community by colonizing various acacia species (*A. gerardii* Benth., *Acacia ehrenbergiana* Hayne, and *Acacia tortilis* (Forssk.) Hayne), along with *T. nilotica* and other xerophytes, such as *L. shawii* and *R. stricta*. Since this habitat is interconnected with wadis, these plants are the common plants in various wadis in desert habitats of Saudi Arabia [35,36,37].

On the other hand, the Qaseem location showed higher evenness and low richness. This location is a wetland, with a water content of 21.56%, that enables specific species (species that flourished in wetlands, i.e., with high water content) to colonize this habitat with a higher number of individuals. The soil analysis showed that the Qaseem location accomplished the highest moisture, clay, silt, salinity, Ca, Mg, Na, K, Cl, SO_4_, HCO_3_, and organic matter. The harsh soil conditions, such as high salinity, can be the factor of low species diversification due to the non-survival of annuals. The inland sabkha of Qaseem forms a brine on the soil, making it difficult for less salt-tolerant plants to grow. Usually, in saline environments, soil factors control plant species’ growth and survival rates, thus, affecting vegetation patterns [38]. Soil factors also reduce plants’ fecundity and germination ability, thus, shaping plant competition and population fitness [39,40]. Most of the dominant and associated species in this location are typical halophytes, such as *C. cretica*, *J. rigidus*, *L. shawii*, and *S. europaea,* which grow in high salt and wet habitats [39,41,42,43], thus, determining the vegetation zonation pattern of salt marshes.

The cluster analysis of the studied locations showed that the northern inland plain of Jouf is dissimilar to the other locations. This could be attributed to the environmental factors, where the soils of the Jouf location showed the lowest salinity. In this location, *T. nilotica* was determined as the dominant species. *T. nilotica* usually colonizes *A. lagopoides* patches, where the altitude is greater than 500 m ASL but grows in soil with low salinity [44].

Among all the regions, the southern coastal sabkha of Jizan has the least diverse vegetation. The soils in this location revealed the lowest moisture content, pH, organic matter, K, sulphate, bicarbonate, calcium carbonate. This could explain the low diversity of this location, as well as the presence of xerophytic plants, such as *C. conglomeratus*, *A. javanica*, and *Z. simplex*.

Overall, the vegetation analysis of the studied location revealed the dominance of *A. lagopoides* in all locations, where it was the most dominant species in the Qareenah, Qaseem, and Salwa locations, and second most dominant species in Jouf and Jizan locations. *A. lagopoides* is one of the most important halophytic grasses in Saudi Arabia. It is a salt excretive grass that grows in the form of patches or mats in highly saline and moistened soil, where it is characterized by structural adaptations and modifications [27,45]. It can tolerate the harsh and saline habitat by expelling the salts it gains, and the plant itself has a very low salt content, making it a palatable forage grass [46]. In addition, this grass has small and waxy leaves, as well as a network of roots and underground rhizomes that help the plant to survive in high salty conditions, even in the summer season, where the salinity becomes even higher [47]. According to our field observation, the *A. lagopoides* has phenotypic plasticity, where its morphology is changed from one location to another. This observation could be a way of adapting to the harsh conditions.

Within the studied locations, other halophytic plants (highly salt-tolerant species) were determined with high abundances, such as *S. aegyptiaca*, *Z. album*, *T. nilotica*, *S. aegyptiaca*, *C. cretica*, and *S. europaea*. These species flourish in saline habitats, where moisture and salinity shaped the community structure [25,48].

## 4. Materials and Methods

### 4.1. Study Area

**Figure 6:**
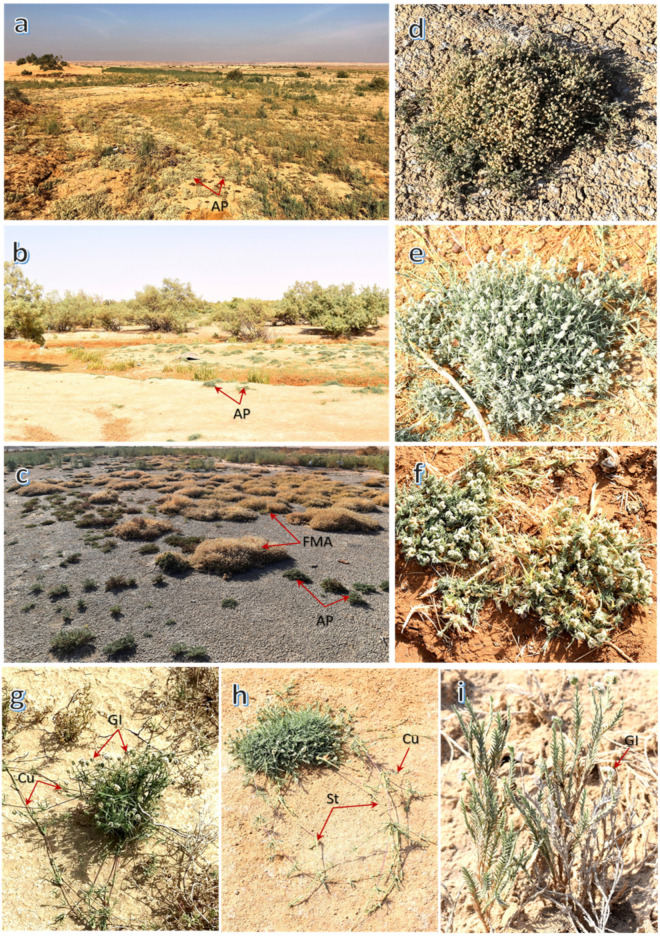
The populations of *Aeluropus lagopoides* (L.) Thwaites in different locations of Saudi Arabia (**a**–**c**), and different morphological growth forms (**d**–**i**). AP: *Aeluropus* patch, FMA: full mature *Aeluropus* patch, Cu: culm, GI: globose inflorescence, St: stolon.

The study was carried out from 2020 through 2021 around the entirety of Saudi Arabia to explore the vegetation zones of the halophyte *A. lagopoides* (Figure 6). *A. lagopoides* mosaic vegetation has been shaped in different eco-regions of Saudi Arabia based on soil properties of the habitat and the morphological adaptation of the indicator plant via phenotypic plasticity.

**Figure 7:**
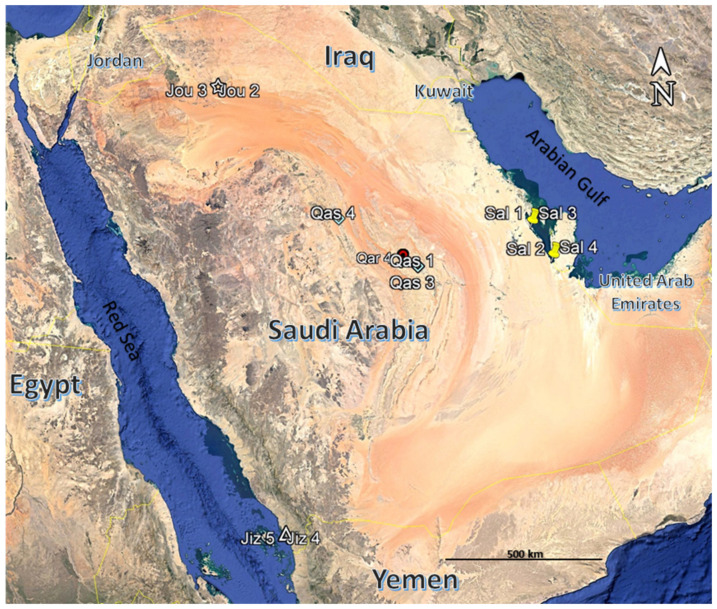
Map of Saudi Arabia showing the different locations of sampled *Aeluropus lagopoides* communities. Qareenah (Qar), Qaseem (Qas), Salwa (Sal), Jouf (Jou), and Jizan (Jiz).

We monitored this grass in five sabkha regions (Figure 7) of Saudi Arabia which represents both coastal and inland sabkhas as follows:

(1) Salwa; coastal sabkha as lowland on the coast of the Arabian Gulf,
(2) Jizan; coastal sabkha on the Southern Coastal Region of Jizan on the Red Sea coast,
(3) Qareenah; inland sabkha in wadi Hargan, Riyadh Region,
(4) Qaseem, inland sabkha of the Al-Aushazia location, and
(5) Jouf; inland sabkha in Domat Aljandal.

The climate of Saudi Arabia is dry-hot and is classified as an arid region occupying about 5% of the world’s arid zones [49]. It has low relative humidity except along the coastal zones, where it sometimes reaches 100%. The mean annual temperature is 33 °C during summer and 14 °C in winter, with a wide seasonal and diurnal variation [50]. The mean solar radiation was recorded highest for July and August and lowest for December and January except in Jizan. However, the rate of pan-evaporation was minimal along the coastal and high mountainous terrain and maximal in the interior due to the maximum presence of desert conditions. High rainfall variations and long drought periods have been recorded between the years without any rain. The climate data were collected between 1999 and 2019 from https://en.climate-data.org/asia/saudi-arabia-29/ (accessed on 15 May 2021) (Figure S2).

### 4.2. Vegetative Sampling

The vegetation sampling for each location was conducted from September to March when *A. lagopoides* was fully growing in each location and was in the full maturity stage. For each location and depending on species growth and form of vegetation composition of the studied area, 10 quadrats were selected randomly from each region (Table 3). The nested quadrat’s area was 10 m × 10 m for shrubs and 5 m × 5 m for small shrubs and herbaceous species. These quadrats were chosen within the sabkha area dominated by patches of *A. lagopoides* population. The species in each quadrat were identified and named according to Chaudhary [35] and Miller et al. [51], as well as following the website http://www.powo.org (accessed on 1 February 2022). The plant density was determined according to Bonham [52], while the plant cover was estimated based on the scale of Braun-Blanquet [53]. To assess species dominance in each location, species importance value was calculated by the summation of relative density and relative cover of each species. The species life forms were identified according to Raunkiaer [54], while the chorotypes of all the species were made to assess the recorded species to World Geographical Groups.

### 4.3. Soil Sampling and Analysis

From each quadrat (n = 10) where vegetative sampling was conducted, three soil samples (0–30 cm depth) were collected from three random positions in plastic bags and pooled as a composite sample. All the soil samples were duly labeled and transferred to Range Science Lab, College of Food Science and Agriculture, King Saud University, Riyadh, Saudi Arabia for further analyses. In addition, a portion of each sample was collected in moisture tins for the determination of soil moisture content by the weight-loss method. The soil samples were spread over separate plastic sheets, air-dried at room temperature, filtered through a 2 mm sieve to remove any debris, if present, and stored in a plastic bag until further analyses. Soil texture for sand, silt, and clay fractions were analyzed by the hydrometer method [55]. Soil organic matter (OM) was determined by wet combustion with dichromate at 450 °C [56]. Soil water extracts (1:5) were prepared for the estimation of soil electrical conductivity (EC) and pH [56]. Soluble inions (Cl and SO_4_) were determined by titration method, while the determination of soluble cations (Ca, Mg, Na, and K), using a flame photometer according to Rhoades [57].

### 4.4. Data Analysis

In this study, multivariate analysis was applied viz. classification and ordination. Based on the data of relative density and cover of all species inside the quadrats (n = 10) of each region, a matrix of species importance values (relative density + relative cover) was constructed and subjected to hierarchical cluster analysis for classification and detrended correspondence analysis (DCA) for ordination using PAST 4.03 software [58]. Species rarefaction for base species richness and abundance in all the studied regions was analyzed using PAST 3X. The soil variables for the studied regions were subjected to one-way ANOVA and the mean values were separated based on Duncan’s test at 0.05 probability level to examine the significant difference among studied regions. In order to detect the relationship between plants (dominant and important plant species with high importance values) of the studied area on one hand and soil variable data, on the other hand, canonical correspondence analysis (CCA) was conducted using MultiVariate Statistical Package (MVSP Version 3.2, Kovach Computing Services, Pentraeth, Wales, UK) according to Ter Braak and Smilauer [59]. In the CCA analysis, two datasets were constructed; one regarding the importance values of the dominant and important species (performed like that for DCA) and the second of the soil parameters of the quadrats (n = 10) of each region. Also, Pearson’s correlation heatmap between the soil variables and the dominant and important species was performed using the XLSTAT software program (version 2018, Addinsoft, NY, USA).

## 5. Conclusions

The present study revealed variance among the community structure of *A. lagopoides,* within different sabkhas in Saudi Arabia. The community of inland sabkhas (Qareenah and Qaseem locations) showed higher plant diversity compared to the coastal sabkhas. The plant diversity of the *A. lagopoides* communities is mainly shaped by the salinity and water content. Moreover, the survival and flourishment of the halophytic grass *A. lagopoides* within a wide soil range in sabkhas revealed the adaptability of this plant to the harsh environment, which could be ascribed to its structural adaptations and modifications, as well as the phenotypic plasticity. Since *A. lagopoides* has many economic potentialities, where it is utilized as fodder, stabilizes sand dunes, used for landscaping of urban areas, the conservation of these natural vegetation zone habitats is of vital importance. Also, this valuable plant could be integrated as a promising forage candidate in saline-affected areas, even in the summer dry season.

## Figures and Tables

**Table 1:** Plant species richness, evenness, and dominance of the studied sabkha locations of Saudi Arabia.

Location	Richness	Evenness	1st Dominant	2nd Dominant	Important Species
Qareenah	2.53	0.90	*Aeluropus lagopoides* (L.) Thwaites (44.41) *	*Zygophyllum coccineum* L. (25.64)	*Juncus rigidus* Desf. (24.14) *Tamarix nilotica* (Ehrenb.) Bunge (12.88) *Rhazya stricta* Decne. (10.79) *Acacia gerrardii* Benth. (10.77) *Phragmites australis* (Cav.) Trin. ex Steud. (9.72)
Qaseem	1.6	0.74	*Aeluropus lagopoides* (L.) Thwaites (94.20)	*Suaeda aegyptiaca* (Hasselq.) Zohary (28.61)	*Cressa cretica* L. (28.23) *Juncus rigidus* Desf. (15.45) *Lycium shawii* Roem. & Schult. (8.97) *Salicornia europaea* L. (6.87)
Salwa	1.04	0.81	*Aeluropus lagopoides* (L.) Thwaites (66.62)	*Zygophyllum album* L.f. (41.05)	*Phragmites australis* (Cav.) Trin. ex Steud. (22.40) *Juncus rigidus* Desf. (15.39) *Suaeda aegyptiaca* (Hasselq.) Zohary (14.95) *Phoenix dactylifera* L. (6.48)
Jouf	1.53	0.75	*Tamarix nilotica* (Ehrenb.) Bunge (74.68)	*Aeluropus lagopoides* (L.) Thwaites (45.61)	*Zygophyllum album L.f.* (33.48) *Cressa cretica* L. (28.80) *Suaeda aegyptiaca* (Hasselq.) Zohary (8.46)
Jizan	1.84	0.75	*Suaeda aegyptiaca* (Hasselq.) Zohary (80.76)	*Aeluropus lagopoides* (L.) Thwaites (54.52)	*Panicum repens* L. (11.23) *Cyperus conglomeratus* Rottb. (11.12) *Aerva javanica* (Burm.f.) Juss. ex Schult. (8.29) *Zygophyllum simplex* L. (8.20)

* represents the importance value based on the relative plant density and cover.

**Table 2:** Soil chemical and physical properties of the studied locations dominated by *Aeluropus lagopoides* community.

Parameter	Location					*p*-Value
	Qareenah	Qaseem	Salwa	Jouf	Jizan	
Moisture %	8.90 ± 1.896 ^b,^#	21.56 ± 3.456 ^a^	5.86 ± 2.439 ^b,c^	4.97 ± 1.005 ^b,c^	1.82 ± 0.424 ^c^	0.0001 ***
pH	8.38 ± 0.18 ^a^	8.39 ± 0.14 ^a^	8.42 ± 0.12 ^a^	8.12 ± 0.11 ^a^	8.07 ± 0.21 ^a^	0.474
Clay %	12.48 ± 0.65 ^b^	16.80 ± 1.26 ^a^	14.20 ± 1.99 ^a,b^	13.24 ± 0.95 ^a,b^	13.87 ± 0.994 ^a,b^	0.188
Silt %	19.28 ± 3.22 ^b,c^	48.40 ± 2.76 ^a^	8.60 ± 1.60 ^d^	26.00 ± 10.77 ^b,c^	42.01 ± 5.272 ^a,b^	0.0005 ***
Sand %	68.24 ± 3.77 ^a^	34.80 ± 2.52 ^c^	77.66 ± 3.23 ^a^	60.76 ± 10.90 ^a,b^	44.11 ± 5.667 ^b,c^	0.0004 ***
EC (dS·m^−1^)	13.02 ± 2.83 ^b,c^	26.30 ± 3.87 ^a^	22.17 ± 7.65 ^a,b^	8.63 ± 3.02 ^c^	9.69 ± 6.76 ^c^	0.003 **
Ca (meq/L)	19.10 ± 2.33 ^b^	39.86 ± 3.74 ^a^	40.07 ± 4.54 ^a^	22.50 ± 2.93 ^b^	24.31 ± 16.96 ^c^	0.003 **
Mg (meq/L)	41.50 ± 10.83 ^b^	69.13 ± 14.74 ^a^	31.18 *±* 12.79 ^a^	15.00 ± 3.23 ^b^	25.76 ± 18.78 ^c^	<0.0001 ***
Na (meq/L)	59.53 ± 17.32 ^a,b^	134.81 ± 33.36 ^a^	132.43 ± 52.52 ^b,c^	37.92 ± 17.24 ^b,c^	42.56 ± 28.836 ^c^	0.001 **
K (meq/L)	10.49 ± 4.46 ^a,b^	20.22 ± 4.97 ^a^	18.25 ± 7.55 ^a^	11.01 ± 8.95 ^b^	4.72 ± 3.366 ^b^	0.02 *
Cl (meq/L)	111.40 ± 25.40 ^a,b^	241.50 ± 40.92 ^a^	211.80 ± 75.15 ^a^	78.80 ± 26.48 ^a,b^	92.62 ± 65.560 ^b^	0.193
SO_4_ (meq/L)	15.64 ± 4.63 ^a,b^	17.78 ± 4.47 ^a^	6.70 ± 1.67 ^b,c^	4.73 ± 3.42 ^c^	0.73 ± 0.048 ^c^	0.007 **
HCO_3_ (%)	3.14 ± 0.20 ^a^	3.58 ± 0.54 ^a^	2.89 ± 0.39 ^a,b^	2.04 ± 0.19 ^b^	0.83 ± 0.229 ^c^	0.0001 ***
OM %	1.63 ± 0.19 ^a^	1.78 ± 0.37 ^a^	0.70 ± 0.09 ^b^	0.67 ± 0.09 ^b^	0.31 ± 0.041 ^b^	0.0001 ***
CaCO_3_%	34.84 ± 2.10 ^a^	15.54 ± 3.38 ^b^	9.60 ± 4.92 ^b,c^	4.16 ± 1.47 ^c,d^	0.55 ± 0.152 ^d^	0.0001***

# Values are mean ± standard errors. EC: electrical conductivity, OM: organic matter. Superscript letters within each row showed significant variation at *p* < 0.05 (Duncan’s test). * *p* < 0.05, ** *p* < 0.01, *** *p* < 0.001 at degree of freedom (*df*) for region (n − 1) = 4 and replications (n − 1) = 9.

**Table 3:** The coordinates and elevation of the different sample quadrats of different locations, Saudi Arabia.

Location	Quadrat No.	Coordinates	Elevation m a.s.l
Qareenah	1	25°03′59.7″ N 46°10′47.7″ E	833
	2	25°03′58.5″ N 46°10′48.1″ E	824
	3	25°03′56.6″ N 46°10′49.4″ E	816
	4	25°03′55.4″ N 46°10′51.1″ E	812
	5	25°03′53.4″ N 46°10′53.8″ E	810
	6	25°03′50.5″ N 46°10′52.9″ E	811
	7	25°03′53.0″ N 46°10′59.6″ E	806
Qaseem	1	26°03′17.7″ N 44°08′10.1″ E	590
	2	26°03′18.5″ N 44°08′15.2″ E	654
	3	26°03′14.2″ N 44°08′13.2″ E	621
	4	26°03′46.2″ N 44°08′16.3″ E	603
Salwa	1	24°45′23.5″ N 50°45′13.5″ E	−10
	2	25°43′39.8″ N 50°08′16.4″ E	−9
	3	25°43′45.5″ N 50°08′02.7″ E	−8
	4	24°45′04.3″ N 50°45′20.9″ E	−11
Jouf	1	29°49′12.0″ N 39°58′23.6″ E	565
	2	29°49′16.2″ N 39°58′27.4″ E	563
	3	29°49′51.0″ N 39°58′55.9″ E	558
	4	29°49′05.1″ N 39°58′08.9″ E	519
Jizan	1	16°58′06.1″ N 42°33′50.9″ E	15
	2	16°58′07.3″ N 42°33′42.4″ E	9
	3	16°58′08.2″ N 42°33′40.0″ E	6
	4	16°58′08.6″ N 42°34′04.9″ E	4
	5	16°58′06.8″ N 42°34′01.0″ E	4

## Data Availability

Not applicable.

## Supplementary Materials

The following supporting information can be downloaded at: https://www.mdpi.com/article/10.3390/plants11050666/s1, Table S1: Floristic analysis of the recorded plant species in the studied sabkha locations of Saudi Arabia, Table S2: Vegetation composition of studied locations dominated with *A. lagopoides* in Saudi Arabia, Figure S1: Species richness and abundance based on the relative density of all the studies regions, Figure S2: Monthly climate data of the surveyed regions.
