# Supplementary material for: Vegetation Composition of the Halophytic Grass Aeluropus lagopoides Communities within Coastal and Inland Sabkhas of Saudi Arabia
Source: Plants (Basel). 2022 Feb 28;11(5):666. doi: 10.3390/plants11050666 (PMC8912373; doi:10.3390/plants11050666)
Supplement: Supplementary file 1 [file plants-11-00666-s001.zip › plants-1570420-supplementary.pdf]

plants

Articles

Vegetation Composition of the Halophytic Grass *Aeluropus lagopoides* Communities Within Coastal and Inland Sabkhas of Saudi Arabia

Basharat M. Dar [1], Abdulaziz M. Assaeed [1], Saud L. Al-Rowaily [1], Abdullah A. Al-Doss [1] and Ahmed M. Abd-El-Gawad [1,2,*]

1 Plant Production Department, College of Food & Agriculture Sciences, King Saud University, P.O. Box 2460, Riyadh 11451, Saudi Arabia; aibrahim2@ksu.edu.sa (A.M.A.-E.); assaeed@ksu.edu.sa (A.M.A.); bdar@ksu.edu.sa (B.A.D.); srowaily@ksu.edu.sa (S.L.A.); aaldoss@ksu.edu.sa (A.A.A.-D.)

2 Department of Botany, Faculty of Science, Mansoura University, Mansoura 35516, Egypt; dgawad84@mans.edu.eg

* Correspondence: aibrahim2@ksu.edu.sa (A.M.A.-E.);±966-599458396.

Supplementary Materials

Citation: Dar, B.A.; Assaeed, A.M.; Al-Rowaily, S.L.; Al-Doss, A.A.; Abd-ElGawad, A.M. Vegetation Composition of the Halophytic Grass *Aeluropus lagopoides* Communities within Coastal and Inland Sabkhas of Saudi Arabia. *Plants* **2022**, *11*, 666. https://doi.org/10.3390/plants11050666

Academic Editors: Robert Philipp Wagensommer and Panayiotis Dimitrakopoulos

Received: 9 January 2022
Accepted: 24 February 2022
Published: 28 February 2022

Publisher's Note: MDPI stays neutral with regard to jurisdictional claims in published maps and institutional affiliations.

Copyright: © 2022 by the authors. Licensee MDPI, Basel, Switzerland. This article is an open access article distributed under the terms and conditions of the Creative Commons Attribution (CC BY) license (http://creativecommons.org/licenses/by/4.0/).

Table S1. Floristic analysis of the recorded plant species in the studied sabkha locations of Saudi Arabia

**Family**	**Botanical name**	**Chorotype**	**LifeSpan**	**Life Form**	**Salt Resistance**	**Location**				
						**Qareenah**	**Qaseem**	**Salwa**	**Jouf**	**Jizan**
Adiantaceae	*Adiantum capillus-veneris* L.	EU-SI-ME-IR-TU	Per	Hem	Gly	√				
Aizoaceae	*Sesuvium verrucosum* Raf.	SA-AR-SU-DC	Ann	Th	Hal					√
	Trianthema portulacastrum L.	AM	Per	Th	Gly					√
Amaranthaceae	*Aerva javanica* (Burm.f.) Juss. ex Schult.	TR	Per	Ch	Gly					√
Apocynaceae	*Rhazya stricta* Decne.	SA-AR	Per	Ph	Gly	√				
Arecaceae	Phoenix dactylifera L.	SA-AR	Per	Ph	Hyphal	√	√	√		
Asclepiadaceae	*Calotropis procera* (Aiton) Dryand.	SU	Per	Ph	Gly					√
Asteraceae	*Eclipta prostrata* (L.) L.	SUB-T	Eph	Th	Gly	√				
	Launaea intybacea (Jacq.) Beauverd	SA-AR	Eph	Th	Gly					√
	Sonchus oleraceus (L.) L.	EU-SI-ME-IR-TU	Ann	Th	Gly	√				
Boraginaceae	*Heliotropium bacciferum* Forssk.	SA-AR-SU	Per	Ch	Gly		√	√		
Chenopodiaceae	*Haloxylon salicornicum* (Moq.) Bunge ex Boiss.	SU	Per	Ch	Gly				√	
	Halocnemum strobilaceum (Pall.) M.Bieb.	ME-IR-TU-SA-AR	Per	Ch	Hal			√		
	Salicornia europaea L.	ME-EU-SI	Ann	Th	Hal		√			
	Salsola incanescens C.A. Mey.	SA-AR	Ann	Th	Hal		√	√		
	Suaeda aegyptiaca (Hasselq.) Zohary	SA-AR	Ann	Hem	Hal		√	√	√	
	Salsola monoica (Forssk. ex J.F.Gmel.) Poir.	SU	Per	Ph	Hal					√
	Suaeda vermiculata Forssk. ex J.F.Gmel.	SA-AR	Per	Ch	Hal			√		
Cleomaceae	*Dipterygium glaucum* Decne.	SU-ZA	Ann	Ch	Xer					√
Convolvulaceae	*Convolvulus pilosellifolius* Desr	IR-TU	Ann	Ge	Gly					√
	Cressa cretica L.	ME-IR-TU	Per	Hem	Hal		√	√	√	
Cucurbitaceae	*Citrullus colocynthis* (L.) Schrad.	SA-AR	Per	Hem	Gly				√	
Cyperaceae	*Cyperus conglomeratus* Rottb.	SA-AR	Per	Hem	Gly			√		√
Ephedraceae	*Ephedra foliata* Boiss. ex C.A.Mey.	SU	Per	Ph	Gly	√				
Fabaceae	*Prosopis juliflora* (Sw.) DC.	SA-AR	Per	Ph	Gly		√			√
	Alhagi graecorum Boiss.	ME-IR-TU	Per	Hem	Hyphal	√	√	√	√	
Frankeniaceae	*Frankenia pulverulenta* L.	EU-SI-ME-IR-TU	Eph	Ch	Hal	√				
Juncaceae	*Juncus rigidus* Desf.	IR-TU-SA-AR	Per	Hem	Hyphal	√	√	√		
Mimosaceae	*Acacia gerrardii* Benth.	SU-AF	Per	Ph	Gly	√				
	Acacia ehrenbergiana Hayne	SU	Per	Ph	Gly	√				
	Acacia tortilis (Forssk.) Hayne	SU	Per	Ph	Gly	√				
	Prosopis farcta (Banks & Sol.) J.F.Macbr.	IR-TU	Per	Ph	Hal	√				
Moraceae	*Ficus salicifolia* Vahl.	ME-IR-TU	Per	Ph	Gly	√				
Poaceae	*Aeluropus lagopoides* (L.) Thwaites	IR-TU-SA-AR	Per	Hem	Hal	√	√	√	√	√
	Cenchrus ciliaris L.	SA-AR	Per	Hem	Gly					√
	Cynodon dactylon (L.) Pers.	PL-T	Per	Ge	Gly	√				
	Dactyloctenium scindicum Boiss.	IR-TU-SA-AR	Eph	Ch	Hal					√
	Panicum repens L.	SUB-T	Per	Ch	Gly					√
	Pennisetum divisum (Forssk. ex J.F.Gmel.) Henrard	SA-AR	Per	Ch	Gly	√				
	Phragmites australis (Cav.) Trin. ex Steud.	PL-T	Per	Ge	Hyphal	√	√	√		
Resedaceae	*Ochradenus baccatus* Delile	SU-AR	Per	Ph	Gly	√				
Solanaceae	*Lycium shawii* Roem. & Schult.	SA-AR-SU	Per	Ph	Gly		√			
Tamaricaceae	*Tamarix aphylla* (L.) H.Karst.	SU	Per	Ph	Hyphal		√			
	Tamarix nilotica (Ehrenb.) Bunge	SA-AR	Per	Ph	Hyphal	√		√	√	
Typhaceae	*Typha domingensis* Pers.	ME-IR-TU-SA-AR	Per	He	Gly	√				
Zygophyllaceae	*Zygophyllum album* L.f.	SA-AR	Per	Ch	Hal	√		√	√	
	Zygophyllum coccineum L.	SA-AR	Per	Ch	Hal	√				
	Zygophyllum simplex L.	SU	Eph	Th	Hal					√

Qar: Qareenah, Qas: Qaseem, Sal: Salwa, Jouf: Jouf, Per: Perrenial, Ann.: Annual, Eph: Ephermal SA-AR: Saharo-Arabian, SU: Sudian, SU-AF: Sudanian-African, SA-AR-SU: Saharo-Arabian-Sudanian, PL-T: Plurireginalbor-trop, ME-IR-TU: Med-Irano-Turanian, IR-TU-SA-AR: Irano-Turanian-Saharo-Arabian, EU-SI-ME-IR-TU: Euro-Siberian-Med-Irano-Turanian, TR-Tropical, SU-ZA: Sudano-Zambezian, SUB-T: Subtropical-Tropical SA-South-American, NT-Neotropical, ME-IR-TU-SA-AR: Med-Irano-Turanian-Saharo-Arabian, ME-EU-SI: Med-Euro-Siberian, IR-TU-SA: Irano-Turanian-Saharo-Arabian, IR-TU: Irano-Turanian, ES-ME-IR-TU: Euro-Siberian-Med-Irano-Turanian, AM-American Life forms: Ch: Chamaephytes, He: Hemicryptophytes, Ph: Phanerophytes, Th: Therophytes, Ge-Geophytes, Gly:Glycophyte, Hal:Halophyte, Hypal: Hydrohalophyte, Xer: Xerophyte.

Table S2. Vegetation composition of studied locations dominated with *Aeluropus lagopoides* in Saudi Arabia.

No	Botanical name	Locations				
		Qareenah	Qaseem	Salwa	Jouf	Jizan
1	*Acacia gerrardii* Benth.	10.77*	-	-	-	-
2	*Acacia ehrenbergiana* Hayne	8.13	-	-	-	-
3	*Acacia tortilis* (Forssk.) Hayne	2.48	-	-	-	-
4	*Adiantum capillus-veneris* L.	6.70	-	-	-	-
5	*Aeluropus lagopoides* (L.) Thwaites	44.42	94.20	66.62	45.61	54.52
6	*Alhagi graecorum* Boiss.	6.22	1.45	1.43	1.68	-
7	*Aerva javanica* (Burm.f.) Juss. ex Schult.	-	-	-	-	8.29
8	*Calotropis procera* (Aiton) Dryand.	-	-	-	-	1.17
9	*Citrullus colocynthis* (L.) Schrad.	-	-	-	3.43	-
10	*Cenchrus ciliaris* L.	-	-	-	-	1.73
11	*Convolvulus pilosellifolius* Desr	-	-	-	-	1.73
12	*Cressa cretica* L.	-	28.23	4.94	28.80	-
13	*Cynodon dactylon* (L.) Pers.	5.24	-	-	-	-
14	*Cyperus conglomeratus* Rottb.	-	-	3.95	-	11.12
15	*Dactyloctenium scindicum* Boiss.	-	-	-	-	2.99
16	*Dipterygium glaucum* Decne.	-	-	-	-	2.82
17	*Eclipta prostrata* (L.) L.	1.46	-	-	-	-
18	*Ephedra foliata* Boiss. ex C.A.Mey.	4.90	-	-	-	-
19	*Haloxylon salicornicum* (Moq.) Bunge ex Boiss.	-	-	-	3.85	-
20	*Heliotropium bacciferum* Forssk.	-	-	6.15	-	-
21	*Halocnemum strobilaceum* (Pall.) M.Bieb.	-	-	6.27	-	-
22	*Ficus salicifolia* Vahl	1.38	-	-	-	-
23	*Frankenia pulverulenta* L.	1.12	-	-	-	-
24	*Juncus rigidus* Desf.	24.15	15.45	15.39	-	-
25	*Launaea intybacea* (Jacq.) Beauverd	-	-	-	-	2.37
26	*Lycium shawii* Roem. & Schult.	5.84	8.97	-	-	-
27	*Ochradenus baccatus* Delile	1.09	-	-	-	-
28	*Panicum repens* L.	-	-	-	-	11.23
29	*Pennisetum divisum* (Forssk. ex J.F.Gmel.) Henrard	5.90	-	-	-	-
30	*Phoenix dactylifera* L.	2.16	-	6.48	-	-
31	*Phragmites australis* (Cav.) Trin. ex Steud.	9.72	4.42	22.40	-	-
32	*Prosopis farcta* (Banks & Sol.) J.F.Macbr.	4.67	-	-	-	-
33	*Prosopis juliflora* (Sw.) DC.	0.00	6.71	-	-	6.56
34	*Rhazya stricta* Decne.	10.80	-	-	-	-
35	*Salicornia europaea* L.	-	6.87	-	-	-
36	*Salsola incanescens* C.A. Mey.	-	0.79	4.97	-	-
37	*Sesuvium verrucosum* Raf.	-	-	-	-	3.48
38	*Sonchus oleraceus* (L.) L.	0.78	-	-	-	-
39	*Suaeda aegyptiaca* (Hasselq.) Zohary	-	28.61	14.95	8.46	-
40	*Salsola monoica* (Forssk. ex J.F.Gmel.) Poir.	-	-	-	-	80.76
41	*Suaeda vermiculata* Forssk. ex J.F.Gmel.	-	-	4.17	-	-
42	*Tamarix aphylla* (L.) H.Karst.	-	4.28	-	-	-
43	*Tamarix nilotica* (Ehrenb.) Bunge	12.88	-	1.25	74.68	-
44	*Trianthema portulacastrum* L.	-	-	-	-	3.04
45	*Typha domingensis* Pers.	2.67	-	-	-	-
46	*Zygophyllum album* L.f.	0.88	-	41.05	33.48	-
47	*Zygophyllum coccineum* L.	25.65	-	-	-	-

48	*Zygophyllum simplex* L.	-	-	-	-	8.20

* values are the average of the importance values of species based on the relative cover and density.

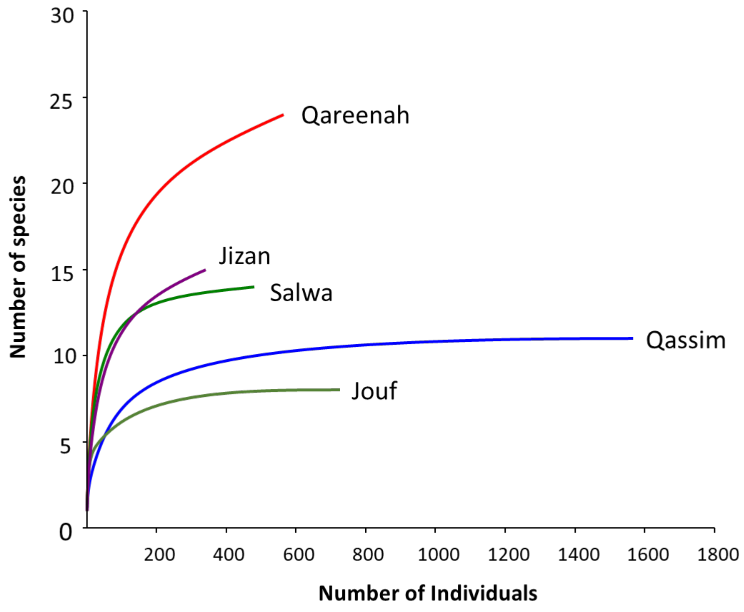

Figure S1. Species richness and abundance based on the relative density of all the studies regions.

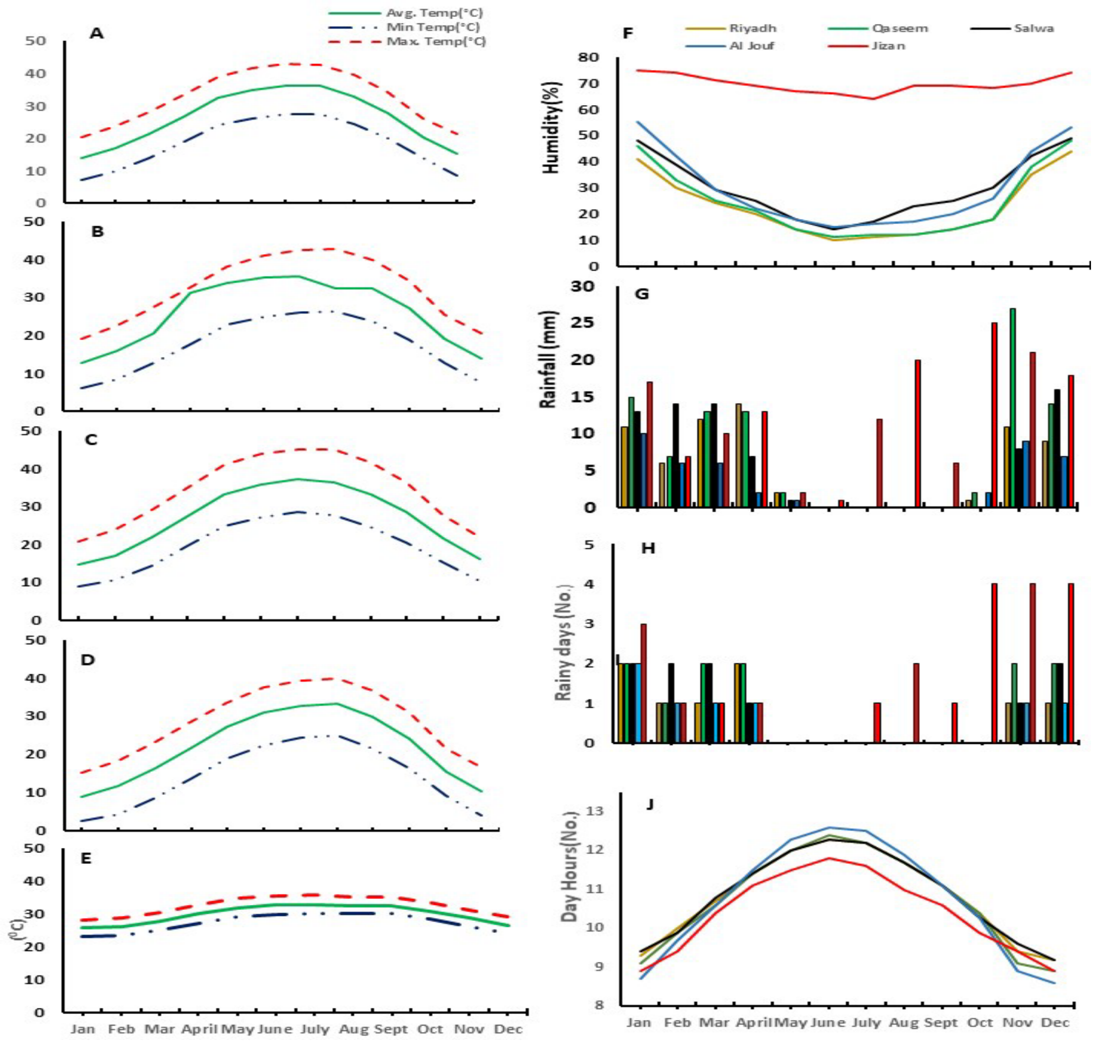

Figure S2. Monthly climate data of the surveyed regions. (A-E) showing max, min, and avg. the temperature of Riyadh, Qaseem, Salwa, Jouf, and Jizan, respectively. (F) Relative Humidity (G) Rainfall (H) Rainy days (J) light duration hours/day. (https://en.climate-data.org/asia/saudi-arabia-29/)
